# A Case Report Highlighting That Silica Gel Products Are Not Always Benign

**DOI:** 10.5811/cpcem.2020.7.47673

**Published:** 2020-10-09

**Authors:** Nolan Lassiter, Nhia Yang, Lakshma Tiyyagura, K. Scott Whitlow

**Affiliations:** * Touro University - California - College of Osteopathic Medicine, Vallejo, California; † Sacramento City College, Stockton, California; ‡ Gastroenterology, Stockton, California; § Touro University - California - College of Osteopathic Medicine, Department of Emergency Medicine, Vallejo, California

**Keywords:** Silica gel, esophageal obstruction, desiccant

## Abstract

**Introduction:**

Silica gel packets are commonly used desiccants for medication products; these packets generally only pose a choking risk in young children. However, new cylindrical desiccant canisters have been developed, which may increase the risk for obstruction in adults.

**Case Report:**

An elderly male presented to the emergency department complaining of difficulty swallowing after taking his morning medications. Through a diligent work-up a desiccant canister was found lodged in the lower esophageal sphincter. The patient was endotracheally intubated and a Roth Net retriever was used to remove the canister.

**Conclusion:**

Cylindrical desiccant canisters pose an increased risk of esophageal obstruction.

## INTRODUCTION

Silica gel products are usually considered minimally toxic substances and primarily pose a risk of choking hazard in young children.^1^ Acute and prolonged oral ingestion of silica is not associated with toxicities of note.^2^ In fact, oral silica gel ingestion is considered so innocuous that it is used in the formulation of solid drugs to serve as a lubricant.^3^ However, this is not to say that silica gel is an entirely benign substance. It has been known to rarely cause silicosis, a fibrotic lung disease that develops due to occupational exposure of respirable silica, with a death rate of 0.74 per one million in 2010.^4^,^5^

**Image 1:**
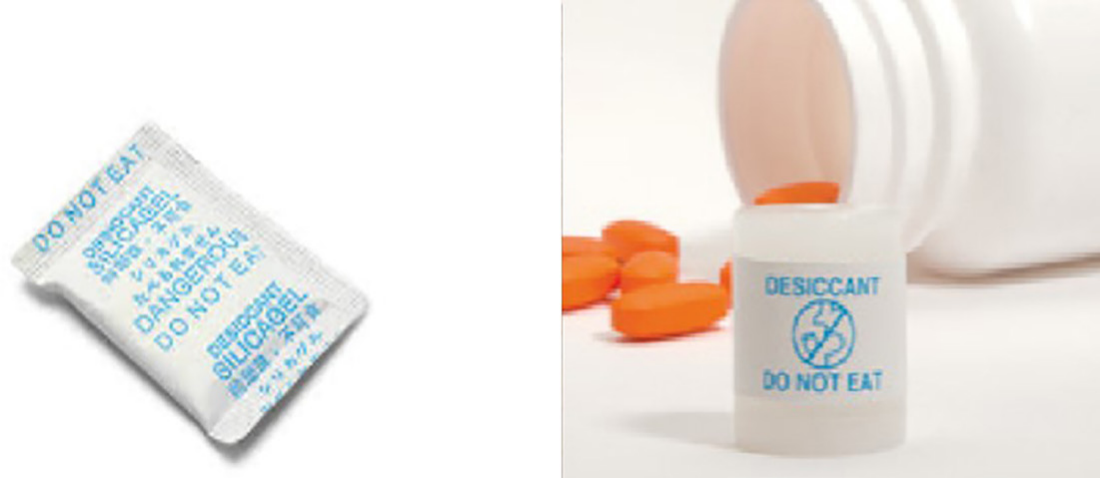
(Left) Image of traditional desiccant package.^1^ (Right) Image of new desiccant cylindrical canister for medications.^7^

Silica gel packets are a common device used as desiccants and are generally made of a paper or cloth that allows the silica to remove moisture from its environment.^2^ The packets serve as such for many over-the-counter and prescription medication products. These packets are frequently ingested by young children, accounting for 2.1% of the annual calls to poison control centers. Fortunately, a vast majority of ingested silica results in an innocuous event, while only occasionally resulting in self-limited mouth and throat discomfort.^6^ Until recently, silica gel has ubiquitously been packaged in paper or cloth packets that have posed a simple choking hazard in young children (Image 1). However, medical device manufacturers have developed a new cylindrical canister that can serve as housing for the silica gel desiccant to be stored with medications (Image 1). These new cylindrical desiccant containers may pose a significant choking hazard in adults. We report a case of a 70-year-old male with a complaint of a foreign body (FB) sensation in his esophagus, chest pain, and an inability to swallow, who unknowingly ingested one of these cylindrical desiccant containers with his daily medications.

## CASE REPORT

A 70-year-old male presented to an urban community emergency department (ED) complaining of substernal chest pain, FB sensation in his esophagus, and difficulty swallowing after taking his multiple, morning oral medications. He stated that he felt as if “something had gotten stuck in his throat” after taking his morning medications. The patient had a past medical history significant for chronic dysphagia, Barrett’s esophagus, congestive heart failure, nystagmus, hyperlipidemia, hypertension, renal insufficiency, and lower esophageal sphincterotomy. After undergoing evaluations for chest pain and epigastric pain, which were negative, he was tolerating per oral (PO) fluids post glucagon and metoclopramide therapy. The ED care team concluded the patient could be discharged safely for out-patient follow-up. A final ambulatory test and a second PO challenge were administered prior to discharge. Upon this PO challenge he reported that he was again unable to swallow PO liquids and had a return of the feeling of “a knot in my esophagus.”

**Image 2:**
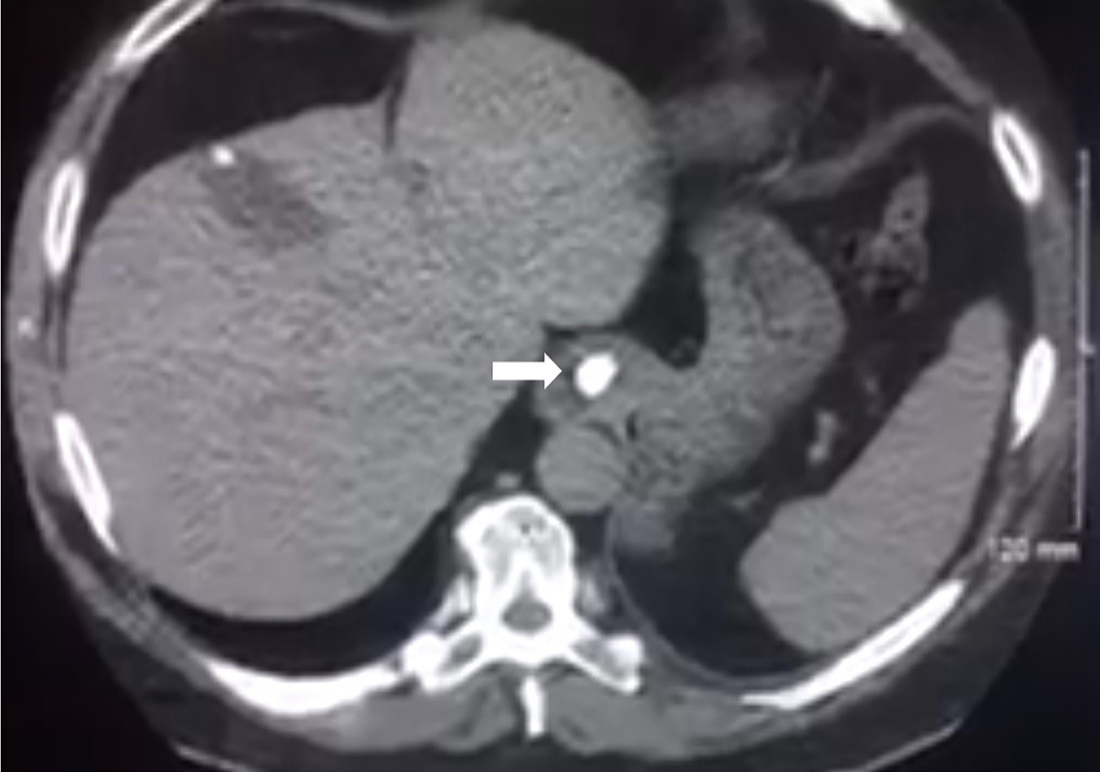
Image of this patient’s computed tomography with the foreign body located near the gastroesophageal junction (arrow).

The discharge was halted, and a non-contrast computed tomography (CT) of the chest was ordered to evaluate the esophagus for stricture or FB. The CT results showed a 15-millimeter (mm) FB located within the gastroesophageal (GE) junction/lower esophageal sphincter (LES) causing a moderately distended and fluid-filled esophagus (Image 2). The ED care team again attempted to mechanically and pharmacologically dislodge the distal FB but was unsuccessful. The fluid in the esophagus was suctioned via insertion of a nasogastric tube to prevent aspiration and to empty the esophagus for possible esophagogastroduodenoscopy (EGD).

CPC-EM Capsule

What do we already know about this clinical entity?

*There is one previously reported incident of a desiccant canister causing esophageal obstruction in a patient with pre-existing esophageal stricture*.

What makes this presentation of disease reportable?

*Obstruction occurred at the gastroesophageal junction in a patient with esophageal stricture*.

What is the major learning point?

*Cylindrical desiccant canisters may easily be mistaken for medications and cause harm in patients with esophageal strictures*.

How might this improve emergency medicine practice?

*Risk of esophageal obstruction has increased in the elderly who take many medications and could easily confuse the canister for a pill*.

**Image 3:**
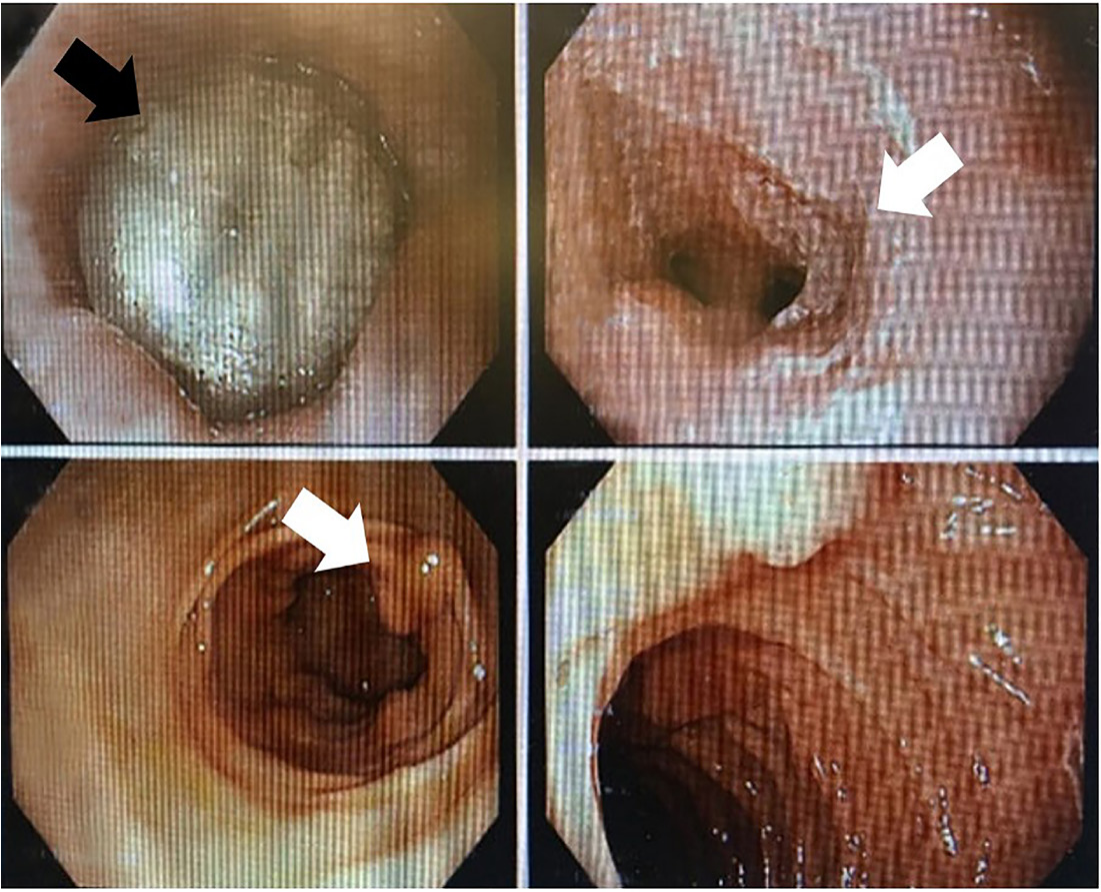
Image of this patient’s esophagogastroduodenoscopy with the foreign body in sight at the gastroesophageal junction displaying the esophageal strictures (white arrows) as well as the trapped desiccant canister (black arrow).

The patient was admitted to the hospitalist service with a gastroenterology consultation for urgent EGD. Upon further inspection with an EGD, the desiccant canister was found in the GE junction along with esophageal strictures (Image 3). The EGD was initially performed under procedural sedation but the endoscopist was also unable to advance the FB through the GE junction with the endoscope alone. The endoscopist then had the patient endotracheally intubated, placed under deep sedation, and performed a second EGD attempt to remove the FB via a Roth Net retriever (STERIS Healthcare, Mentor, OH), which was successful. The FB, after successful removal, was determined to be a cylindrical silica gel canister measuring 11.5 mm. There were no complications secondary to the EGD, deep sedation, intubation, or removal of the FB. The patient tolerated the procedure well, was extubated quickly post EGD and was discharged home after recovery with instructions to follow up with the gastroenterologist as an outpatient. He was also educated to avoid taking multiple medications at once without identifying them.

## DISCUSSION

These relatively recently deployed, cylindrical, silica gel containers, such as the one in this case, are filled with desiccants like bentonite clay and silica gel, with a purpose of moisture absorption to keep products dry and control odors. This patient presented with an esophageal obstruction caused by accidental ingestion of a cylindrical silica gel canister while administering his own medications. He had a history of a known high-grade esophageal stricture, making the risk of esophageal FB obstruction higher and the potential retrieval process even more challenging. In such a case, any patient with any form of esophageal stricture and a retained esophageal FB will require endoscopic retrieval to remove it, if medical management fails.^10^ The clinical decision to implement the use of the Roth Net retriever to remove the FB was based on the failure of simple advancement of the FB into the stomach, the patient’s history of esophageal stricture, and radiographic (CT) imaging providing the precise location and size of the FB.^8^ The retrieved FB was an 11.5-mm cylindrical, silica gel container. The patient did well without any complications after the procedure.

A similar obstruction by a desiccant canister in a patient with pre-existing esophageal strictures was reported in 2015; however, in that case the canister became lodged in the esophagus unlike in our case where the canister was lodged in the LES and GE junction.^10^ As evidenced in this case report and the previously reported literature, the new cylindrical desiccant canisters pose an increased risk of esophageal obstruction in patients with high risk of esophageal obstruction, such as pre-existing esophageal strictures. As patients are frequently required to take many medications daily, it should not be surprising that one could inadvertently ingest a desiccant canister that may be the same color and relative shape of medications themselves, especially if the patient is older with decreased visual acuity.

These desiccant containers appear to have been designed for efficiency of manufacturing and insertion into bottles or other containers. A pamphlet from Clariant, a chemical company that specializes in helping medical device companies with production as well as creating chemicals and polymers used in medical devices such as silica desiccant canisters, describes the canisters as having an “[i]nnovative design and construction [that] allow[s] these components to be inserted automatically at high rates of speed.”^7^ Additionally, the product sheet for the rigid desiccant canisters (for high-speed automatic insertion) describes the product as being “cost-efficient” and having a “robust design.”^7^ All these descriptions place an emphasis on production efficiency and cost reduction.

We attempted to obtain the drug master file to evaluate the pre- and post-market experience with these containers. However, those attempts were unsuccessful. It appears, via our review of the literature regarding these devices, that desiccant companies have attempted to design various fail-safes within the desiccant product design, such as changing the “do not eat” label from blue to a bright red to potentially decrease the risk of mistaking the canister for medication,^11^ but medications come in all colors and, in many cases, similar shapes and sizes. These visual factors, in tandem with the canister’s rigid and non-malleable design in comparison to traditional desiccant packets, increases the risk of obstruction and subsequent complications that may increase morbidity in older, at-risk populations.

## CONCLUSION

Cylindrical desiccant canisters pose an increased risk of esophageal obstruction in patients with high risk of esophageal obstruction, such as a pre-existing esophageal stricture. The transition from paper/cloth desiccant packaging to a non-malleable cylindrical canister may improve production efficiency and cut costs but has unintentionally increased the risk profile of silica gel, which was previously considered an innocuous substance.
